# Untangling the crosstalk between BRCA1 and R-loops during DNA repair

**DOI:** 10.1093/nar/gkab178

**Published:** 2021-03-23

**Authors:** Marta San Martin Alonso, Sylvie M Noordermeer

**Affiliations:** Leiden University Medical Center, Department of Human Genetics, Leiden, The Netherlands; Oncode Institute, Utrecht, The Netherlands; Leiden University Medical Center, Department of Human Genetics, Leiden, The Netherlands; Oncode Institute, Utrecht, The Netherlands

## Abstract

R-loops are RNA:DNA hybrids assembled during biological processes but are also linked to genetic instability when formed out of their natural context. Emerging evidence suggests that the repair of DNA double-strand breaks requires the formation of a transient R-loop, which eventually must be removed to guarantee a correct repair process. The multifaceted BRCA1 protein has been shown to be recruited at this specific break-induced R-loop, and it facilitates mechanisms in order to regulate R-loop removal. In this review, we discuss the different potential roles of BRCA1 in R-loop homeostasis during DNA repair and how these processes ensure faithful DSB repair.

## INTRODUCTION

**Figure 1.**
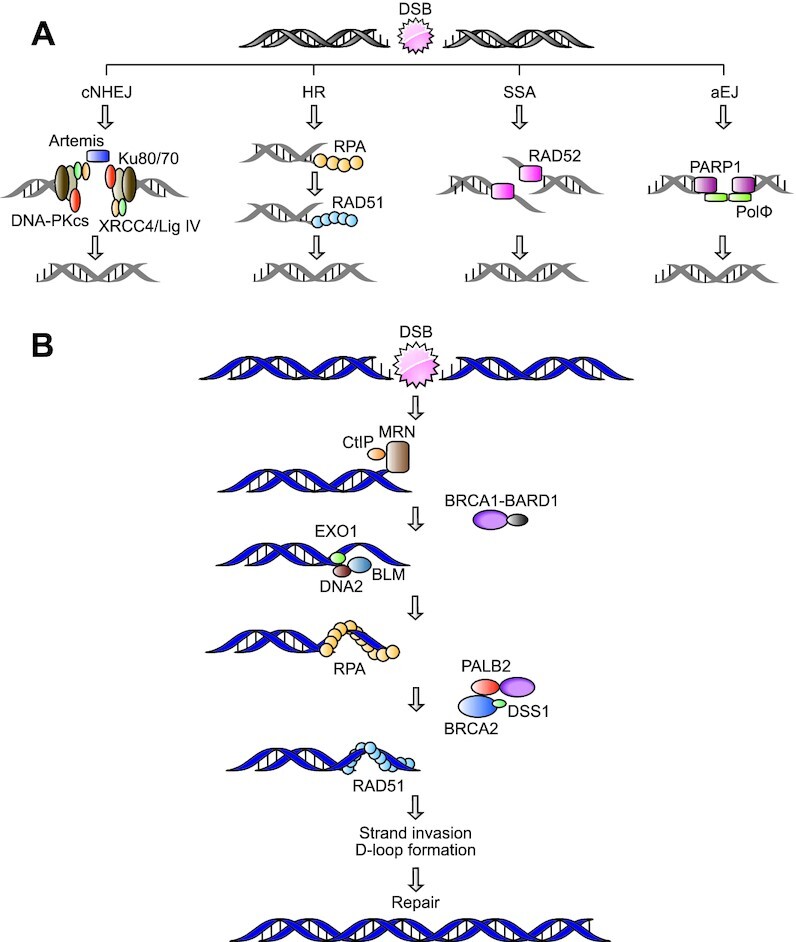
(**A**) Different repair pathways for DSB lesions. Cells exhibit different pathways to respond to the deleterious DSB lesions. Canonical non-homologous end joining (cNHEJ) mediates the ligation between both broken ends in a low fidelity manner. During homologous recombination (HR), cells use a homologous sequence as a template, repairing the break in an error-free manner. Single strand annealing (SSA) uses longer stretches of homologous sequences *in cis* for repair whereas alternative end joining (aEJ) uses microhomologies, both resulting in inaccurate repair. (**B**) HR and its associated proteins. In S-G2 phase, cells can respond to toxic DSBs by means of HR. In this faithful pathway, the MRN complex together with CtIP initiates short-range resection of the 5' ends at both sides of the broken DNA. DNA2 nuclease and the interacting BLM helicase or EXO1 nuclease generate long filaments of ssDNA. This filament is then coated by RPA protein, which is rapidly exchanged by RAD51 recombinase with the help of BRCA2, in complex with BRCA1 and PALB2. This permits strand invasion of the sister chromatid to give rise to the D-loop needed for DNA synthesis and termination of the repair process.

DNA double-strand breaks (DSBs) are one of the most harmful lesions that occur in the DNA. They can be formed due to the action of exogenous agents (such as ionizing radiation (IR), chemical compounds or UV light) or endogenous processes (metabolic reactive oxygen species, replication errors, etc.). The correct repair of DSBs is crucial for the maintenance of genetic integrity and therefore, cells have developed a complex repair machinery known as the DNA damage response (DDR) to overcome them (1). Different pathways exist to repair a DSB: homologous recombination (HR), classical non-homologous end joining (cNHEJ), alternative end joining (aEJ) and single strand annealing (SSA) (Figure 1A). HR is generally considered error free since it uses the intact sister chromatid as a template for repair. Therefore, it is restricted to S-G2 phases of the cell cycle. cNHEJ, aEJ and SSA on the other hand, are prone to induce mutations due to insertion or deletion of genetic material (2). To ensure genetic stability, the cell must decide on the pathway for repair once a DSB occurs in the DNA.

As a first step in DSB repair, H2AX is rapidly phosphorylated at ser139 (γH2AX). This phosphorylation appears immediately after damage induction and spreads to both sides of the break in an asymmetric way (3,4). The spreading of γH2AX acts as a DDR recruitment platform by engaging downstream factors involved in signalling and repair. A crucial step that decides between cNHEJ and the other repair pathways is DNA end resection. 53BP1 prevents resection by recruiting additional cNHEJ factors such as RIF1 and the Shieldin complex, thereby blocking the recruitment of nucleases needed for resection (5). During cNHEJ, DSBs are bound by the KU70/KU80 complex and via minimal end processing mediated by proteins such as Artemis, breaks are simply re-ligated by the XRCC4/Lig4 complex (6).

During HR, DSBs are detected by the MRN complex (composed of MRE11, RAD50, NBS1), which together with CtIP starts the resection of the 5' ends generating 3' single-stranded DNA (ssDNA) overhangs. CDK-dependent phosphorylation of CtIP during S phase enables binding to BRCA1, which enhances MRN-mediated resection and simultaneously inhibits the recruitment of RIF1 (7–10). Subsequently, either EXO1 or DNA2 together with BLM further processes the 5' ends to generate long stretches of 3′ ssDNA, which are rapidly covered and protected by the trimeric RPA complex (RPA1–RPA2–RPA3) (11). BRCA2, associated with DSS1 (12), promotes the loading of the recombinase RAD51 on the ssDNA filament through interactions with PALB2 and BRCA1 (see Figure 1B). The binding of RAD51 to ssDNA forms a presynaptic filament driving the invasion of the ssDNA into the undamaged homologous sequence. This heteroduplex is termed a displacement-loop (D-loop) (13), and biochemical assays have shown that the BRCA1–BARD1 complex binds this structure with high affinity and enhances RAD51 recombinase activity (14). However, whether this stimulating role of BRCA1–BARD1 is directly mediated via its D-loop binding capacity has to be functionally proven.

Next to cNHEJ and HR, two other repair mechanisms can mend a DSB, albeit at the expense of fidelity. During aEJ, DSB ends with microhomologies are joined, leading to deletions and templated insertions (15). SSA requires extensive resection to reveal homologous sequences *in cis* within the same DNA molecule resulting in large deletions (16). Signatures of these pathways are abundant in the human genome, indicating that they might have played a role in evolution.

Many processes regulate DSB repair, including cell cycle, break architecture, and chromatin environment. In the last decade, R-loops—a type of RNA–DNA hybrid—have emerged as important factors in DSB processing and repair. In this review, we will focus on the role of R-loops in genomic stability and their link to BRCA1-mediated HR.

## R-LOOP HOMEOSTASIS

### Normal physiology

An R-loop is a nucleic acid structure consisting of an RNA:DNA hybrid and the associated displaced ssDNA from the original DNA:DNA duplex (17,18). These structures are naturally occurring in the DNA and are able to regulate diverse cellular processes. Yet, they are also considered potential sources of genomic instability when assembled out of their natural context, linking them to recombinogenic DNA damage.

RNA:DNA hybrids are required for several physiological processes. For example, small RNA:DNA hybrids are formed during replication at the start of leading strand synthesis and Okazaki fragment synthesis. Also, the telomeric repeat-containing RNA elements (TERRA) form natural RNA:DNA hybrids with telomeric DNA to enhance chromosome end maintenance and prevent telomeric shortening (19,20). A process demanding R-loop formation is Class Switch Recombination (CSR) of immunoglobulins. In this process, transcription and R-loop formation between repetitive DNA regions called switch (S) activates the enzyme AID. AID deaminates dC into dU in the ssDNA strand of the R loop, promoting DSBs that will be repaired by cNHEJ resulting in class switching (21). Research studying the immunoglobulin heavy-chain (*IgH*) locus has shown that the R-loop at the S region results from the RNA helicase activity of DDX1 upon binding to G-quadruplexes (G4s: structures of stacked guanine tetrads) in the intronic switch RNA (22).

### Unscheduled R-loop formation

Formation and accumulation of unscheduled R-loops can be a source of genetic instability due to the presence of an exposed ssDNA, which is susceptible to mutagenesis and damage (23). Furthermore, an R-loop can create an obstacle for the different machineries that utilize DNA, such as transcription or replication. During the transcription process, any secondary structure, bound protein or lesion in the template can form a roadblock for RNA polymerases (RNAP) (24). In addition to direct RNAP blockage, R-loops can also form an obstacle for replication fork progression giving rise to transcription-replication conflicts (TRCs). TRCs have been widely reviewed elsewhere (25–27). Evidence in yeast has shown that head-on (HO) conflicts (in which both machineries travel in opposite, convergent directions) are more deleterious and cause higher recombination levels than co-directional encounters (CD, in which replication and transcription travel in the same direction) (28).

More recently, it has been shown that the orientation of the conflict influences the level of R-loop accumulation in plasmid systems (29). The study, carried out in human cells using an episomal system, showed that HO conflicts enhance the formation of R-loops due to the increased topological stress that promotes the probability of hybrid formation, whereas CD collisions would help to resolve R-loops (29). Indeed, in bacteria it has been shown that HO conflicts result in pervasive R-loops, inhibiting replication and transcription or even leading to cell death when left unresolved (30).

Despite the above-described data, it is still unclear whether the R-loop itself is the cause or the consequence of the TRCs and whether the physical roadblock that a TRC poses on the replication process is the direct cause of genomic instability. Overexpression of RNase H1—a nuclease specific for cleavage of RNA in RNA:DNA hybrids—led to decreased recombination levels in yeast, pointing to a causal relationship between R-loops and genetic instability (31).

A recent study in yeast observed that R-loop formation occurred in both CD and HO orientations, while only the HO conformation resulted in DNA damage and genetic instability. Yet, stabilization of the R-loops in CD orientation by overexpression of the RNA-binding protein Yra1 also led to increased recombination frequencies (32). Importantly, previous data from the same group indicated that the increased R-loop formation and genomic instability observed upon Yra1 overexpression is transcription dependent and leads to replication defects (33). Taking these data together, the authors hypothesize that under normal conditions, ongoing replication forks can resolve R-loops in the CD orientation, preventing replication defects and genomic instability. The data also suggest that the R-loop is a cause rather than a consequence of the TRC and the accompanying genomic instability. However, these hypotheses have not been proven experimentally.

In yeast, it has been shown that cells carrying specific histone mutants accumulate R-loops that do not compromise the genetic stability by themselves. These mutant strains did not show a cell cycle or transcription defect, indicating that it is not a lack of TRCs that can explain the phenotype, although the authors did not specifically address this. Here, it was postulated that a second step is required to induce instability, likely related to chromatin changes (34).

Also other non-B secondary DNA structures, such as G4 structures, can pose roadblocks to the replication and transcription machineries (35). Importantly, helicases involved in resolving G4 structures, such as PIF1, FANCJ and BLM, are essential gatekeepers of genomic stability (36). In a recent study, human cells treated with specific ligands that stabilize G4 structures showed more R-loops and DNA damage accumulation (37). Indeed, it has been shown previously that G4 structures are formed favourably in coding strands opposite transcribed strands with high R-loop levels, indicating a clear relationship between the two structures (35,38). Altogether, these studies show that R-loops can have harmful consequences on the genome, although it remains speculative what process creates the damage.

### Prevention of R-loops

There is much evidence showing that cells have evolved mechanisms to prevent or remove unscheduled R-loops in order to maintain genomic stability. In this review, we differentiate two strategies: prevention and direct resolution. Different mRNA binding proteins (RBPs) are capable of preventing hybridization of the recently transcribed RNA strand with the DNA used as template for transcription thus impeding hybrid formation (39). One of the first reports linking RBPs to RNA:DNA hybrids showed that mutations in the evolutionary conserved THO complex lead to genetic instability in an R-loop-dependent manner in yeast (31). This complex is involved in correct mRNA ribonucleoprotein complex (mRBP) formation, transcription elongation, and mRNA export (40). Specifically, for the coupling of transcription elongation with mRNA export, THO interacts with TREX, a protein complex formed by the proteins Yra1/ALY and Sub2/UAP56 (41). Several studies have focused on specific members of the THO complex, e.g. Hpr1 in yeast, its counterpart THOC1 in human cells, or the protein THOC-2 in *Caenorhabditis elegans*. These studies showed that the lack of these factors leads to increased R-loop-associated genetic instability observed as high rates of recombination frequency and accumulation of γH2AX or 53BP1 DNA damage foci (42–44). Notably, these instability phenomena were reduced by RNase H1 overexpression *in vivo* and increased by AID overexpression, indicating direct involvement of R-loops to the phenotype (42,45). Depletion of the THO complex in human cells results in transcription elongation defects and impaired replication, supporting the idea that increased R-loop accumulation leads to replication defects (42). More recently, a crosstalk between THOC1 and the SIN3A histone deacetylase complex has been described, suggesting a connection between R-loop homeostasis and chromatin remodelling (46). In this study, the authors observed that the interaction between the THO and SIN3A complexes was necessary to maintain the correct levels of acetylation in order to prevent the accumulation of harmful RNA:DNA hybrids. Given that the lack of both SIN3A or THOC1 led to R-loop accumulation and genetic instability, these data suggest that harmful R-loops are formed in specific chromatin states. Increased R-loops have also been observed in the absence of other RNA binding and processing factors, such as Med13, Sin3 and Bur2 that participate in the suppression of R-loop-mediated gross chromosomal rearrangements (GCR) in yeast cells (47).

Defects in the correct export of mRNA, such as Mlp1 or Mlp2 mutants that affect the nucleopore structure, also lead to higher genetic instability in an R-loop-dependent manner (48). Along the same line, it has been shown that proteins involved in mRNA cleavage and polyadenylation (mCP) (49) or splicing factors (50) are also contributing to control hybrid accumulation. It is an interesting hypothesis that R-loops form differently depending on the cellular defect. Although transcription is the indispensable condition for its formation and there are many reports confirming that higher transcription levels correlate with more R-loop accumulation (51,52), this is not always directly linked. It seems plausible that a defect in RNA elongation and processing leads to R-loop formation because of a higher probability of the elongating RNA strand to hybridize instantly with the surrounding template DNA (31,50). Contrastingly, in those situations where the deficiencies are associated with RNA degradation and export (53), R-loops could form because of an aberrant accumulation of mRNA molecules in the nucleoplasm which increases the chances of *de novo* hybrid formation.

DNA topoisomerase enzymes have an important role in R-loop prevention. These enzymes function in resolving the torsional stress that occurs during transcription and replication. Both Top1 and Top2 can relax positive and negative supercoils in the DNA. Negative supercoiling increases the probability of the newly synthesized RNA to hybridize with the template strand, forming an R-loop behind the transcription machinery. In yeast, R-loops accumulate in the highly transcribed ribosomal DNA (rDNA) to prime origin-independent replication within these regions (54). Loss of topoisomerases exacerbated the R-loop accumulation in these regions, leading to a decrease in transcription (55). Also in human cells, the lack of several proteins involved in DNA metabolism increased R-loop formation. For instance, the absence of TOP1 promoted stalled forks and DNA breaks in specific genes transcribed during S phase. Importantly, prevention of R-loop formation by overexpression of RNaseH1 suppressed these phenotypes (56). Also in bacteria, it has been described that genomic instability phenotypes provoked by the lack of Topoisomerase 1A were R-loop-dependent (57).

### Cellular mechanisms for removal of R-loops

Apart from the above described avoidance mechanisms, cells also possess factors to actively remove R-loops. Monomeric RNase H1 and RNase H2, composed of the three subunits RNase H2A, B and C in eukaryotes, are enzymes that can specifically cleave the RNA strand in the hybrid. In addition to R-loop removal, RNase H2 is also involved in the removal of misincorporated ribonucleotides in DNA. Dysfunction of either RNase H1 or H2 leads to genomic instability, although RNase H2 seems to be responsible for the majority of R-loop removal (58). Additional data has shown that RNase H2 is required specifically for the removal of R-loops post-replication, while RNase H1 is mainly acting on R-loops upon stress induction (59). Apart from these enzymes, many studies have found evidence that the absence of different helicases causes an accumulation of R-loops. Some helicases are able to translocate along the RNA strand whereas other types might not translocate and only act locally (reviewed in (60)). In the current model, helicases resolve DNA:RNA hybrids by unwinding the double stranded structure, although not all of them have been shown to remove hybrids *in vitro*. Examples of helicases for which this activity has been proven are the yeast helicase Pif1 (61), the human proteins DHX9 (62,63), and Senataxin (64–66). The helicase Senataxin (encoded by the gene SETX) or its ortholog Sen1 in yeast, has an important role in the R-loop dissolution (67). *In vitro* studies of purified Sen1 have shown that this protein presents 5′ to 3′ translocation activity along DNA and RNA, as well as 5′ to 3′ unwinding properties (64–66). The deletion of the yeast Sen1 gene induced a genome-wide R-loop accumulation (51) and led to higher transcription-dependent recombination frequencies and DNA damage foci, presumably because of the R-loop accumulation (68). More details regarding this protein will follow.

Another possibility for cells to eliminate R-loops during replication is via dissolution aided by replication-associated repair proteins such as Fanconi anaemia (FA) pathway proteins. The FA genes encode a plethora of >20 proteins involved in resolving interstrand crosslinks (ICLs) and promoting accurate replication. Schwab *et al.* showed that R-loops are resolved by the translocase activity of FANCM (69). Along the same line, lack of FANCD2 or FANCA led to R-loop-induced DNA damage accumulation (69,70). Several groups have reported that the lack of BRCA1 or BRCA2 also leads to accumulation of R-loops (71,72). Bhatia *et al.* used powerful tools such as immunoprecipitation of RNA:DNA hybrids (DRIP) with the specific S9.6 antibody (73), chromatin immunoprecipitation (ChIP), and FACS analysis of the GFP-fused hybrid-binding domain of RNase H1 to show an accumulation of R-loops in general and at specific genomic loci upon BRCA1- and BRCA2-depletion (71). Importantly, BRCA2 was described to interact with the TREX-2 subunit PCID2, involved in RNA biogenesis and export. These observations identified a new role for BRCA2 in R-loop resolution at transcribed regions (71). Indeed, BRCA2 absence promoted the accumulation of R-loops during transcription elongation at specific promoter-proximal pause sites (PPP sites), which gave rise to genetic instability (72). In the next paragraphs, we will provide more details about the role of BRCA1 in R-loop processing.

## BRCA1: SAFEGUARD OF THE GENOME

### The role of BRCA1 in DSB repair

The genomic region 17q21 containing the Breast cancer type 1 susceptibility gene (BRCA1) was first linked to hereditary breast cancer in the early 1990s (74). We now know BRCA1 is a tumour suppressor gene, given that tumour formation in carriers of heterozygous loss-of-function mutations is frequently driven by loss of the wild type allele (75). BRCA1 is primarily associated with familial breast and ovarian cancer, but mutations in this gene also give rise to other familial or sporadic cancer types and disorders, such as prostate cancer (76) and Fanconi Anemia (77).

BRCA1 is a large protein of 1863 amino acids that participates in multiple cellular activities through specific interaction with a large variety of partners. Mouse and human BRCA1 proteins share a 60% identity, showing protein conservation across species (78). BRCA1 not only plays a role in DNA damage repair but also acts in other cellular activities such as cell cycle regulation, chromatin remodelling, replication fork protection, transcription regulation and apoptosis (79). Although BRCA1 has been widely studied, its mechanism of action, as well as the regulation of its interactions during these many processes, remain incompletely understood.

**Figure 2.**
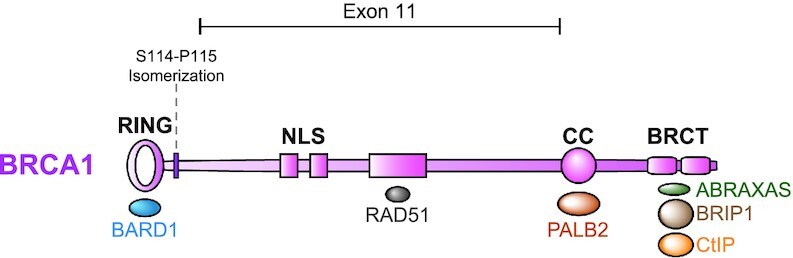
The BRCA1 protein comprises different functional domains. The N-terminal part of BRCA1 contains the RING domain, important for the formation of the heterodimer with BARD1 and its E3 ubiquitin ligase activity. The N-terminus also contains residues involved in PIN1-mediated isomerisation during the replication stress response. The coiled coil (CC) mediates the interaction with PALB2, essential for the binding with BRCA2. A tandem BRCT domain is present at the C-terminal tail of BRCA1, critical to establish the phospho-dependent interaction with ABRAXAS, BRIP1 and CtIP. Apart from these partners, BRCA1 also interacts with other proteins, being involved in multiple functions. Exon 11 comprises more than 60% of the coding sequence of BRCA1, as indicated.

As indicated in Figure 2, BRCA1 comprises multiple protein domains that can interact with different partners. With its N-terminal RING domain, BRCA1 dimerizes with BARD1 (80) and multiple studies have indicated the obligated formation of this heterodimer for its function (81,82). Although mutations in the BRCA1 RING domain occur frequently in tumours (83,84), there are conflicting data on the importance of the RING domain for the recruitment of the heterodimer to specific DNA lesions (85–87) and whether the E3 ligase activity of BRCA1 is involved in tumour suppression. Studies in mouse models with epithelial-specific expression of a C61G BRCA1 RING mutation, which inhibits the interaction between BRCA1–BARD1 and abrogates the E3 ubiquitin ligase activity of the heterodimer, showed that these mice acquired breast tumours at a comparable rate as mice with epithelial BRCA1-deficiency (88). However, the C61G mutation was able to restore therapy resistance unlike the BRCA1-deficient mice. In contrast, Shakya *et al.* did not show increased tumour formation (nor therapy sensitivity) in a mouse model carrying a I26A mutation that impairs the ubiquitin ligase activity but not the heterodimer assembly (89,90). This conclusion was supported by experiments performed in mouse embryonic stem cells (ES cells) in which the mutation I26A in BRCA1 presented a very similar phenotype compared to wildtype cells in terms of HR function and resistance to genotoxic stress (91). In contrast, the Morris lab showed that expression of the same I26A mutation cannot restore resistance to treatment with PARP inhibitors (PARPi) in HeLa cells (92). Furthermore, the mutation R99E in BARD1, which also impairs the E3 ubiquitin ligase activity of the heterodimer, induced sensitivity to a subset of DNA damaging agents, including PARPi. These data indicate that the E3 ligase activity of the BRCA1-BARD1 complex is required for maintenance of genomic stability (92). Opposed to this, others have shown that the R99E BARD1 mutation can fully rescue PARPi sensitivity of BARD1-deficient cells, albeit using a different cell line (86). These contradictory observations warrant that more studies are needed to clarify the role of the E3 ubiquitin ligase activity of BRCA1 in tumour suppression and therapy response.

Independent of its E3 ligase function, the BRCA1-BARD1 dimer also associates directly with the recombinase RAD51, essential for both HR (93) and replication fork protection (92,94). The Sung laboratory described the biochemistry of this complex, identifying mutations that perturbed the interaction with RAD51 affecting the formation of the D-loop and DSB repair by HR (14). In addition, Daza-Martin *et al.* described how a conformational change of the heterodimer BRCA1-BARD1 carried out by the isomerase PIN1 enforces the interaction with RAD51, specifically during replication fork protection (Figure 2) (94).

The C-terminus of BRCA1 contains two copies of BRCT (BRCA1 C-terminal) domains (Figure 2) (95). The BRCT domains form a platform for the phospho-specific recognition of other proteins (96). They have been identified in proteins involved in DNA damage repair, RNA processing, and cell cycle checkpoint. These domains can not only interact with other proteins but also with DNA or poly-ADP-ribose (PAR) (97). BARD1, also bearing two BRCT domains at its C-terminus, has been shown to be critical for the mobilization of the heterodimer to DNA damaged sites by recognizing PAR molecules at DNA lesions, regardless of the H2AX status (98). Recently, it has been reported that BARD1 was able to bind ubiquitylated H2A at lysine 15 directly through its BUDR (BRCT domain ubiquitin-dependent recruitment) motif and this occurred synergistically with the binding of its ankyrin repeat domain (ARD) to H4K20me0, generating a strong interaction between damaged chromatin and BARD1 in S and G2 phases (87).

Mutations in the BRCT domains of BRCA1 have been detected frequently in cancer patients. Intact BRCT domains are important for the formation of the BRCA1-A (with ABRAXAS, RAP80, BRE, BRCC36 and MERIT40), -B (with BACH1/BRIP1/FANCJ) or -C (CtIP) complexes, regulating HR (99). BACH1 is a DEAH helicase required for DNA repair (100). Together with CtIP, BRCA1 stimulates the initial short-range DNA end resection at the DSB site and is also involved in regulating the speed of resection (8). In addition to simple DSB end resection, BRCA1 and CtIP also show a role in the resection of DNA with G4 structures. BRCA1 shows high affinity for these structures and brings its interacting partner PIF1 to these sites. This protein is necessary for the unwinding of unusual DNA conformations to facilitate resection and HR (101).

Interestingly, the BRCA1-A complex has been reported to inhibit HR by controlling excessive DNA end resection. The disruption of this complex leads to an increase in sister chromatid exchange, higher sensitivity to genotoxic agents, and chromosome aberrations (102,103), suggesting that the correct balance of HR for repair is required for genetic integrity. The central part of *BRCA1* is encoded by exons 11–13 and contains a coiled-coil (CC) domain that mediates the interaction with its partner PALB2 (Figure 2) (104). PALB2 acts as a bridging protein by mediating the interaction of BRCA1 with BRCA2 and is essential for RAD51 loading during HR (105).

Many *BRCA1* mutations in tumours arise in exon 11, the largest exon of the gene encoding for ∼60% of the protein (Figure 2). Many of these mutations are known to induce the expression of a specific splice variant of BRCA1, lacking exon 11 (106,107). This variant retains the RING domain, CC domain, and the BRCT motifs, but lacks two nuclear localization signals (NLSs). However, it displays a hypomorphic function showing only partial RAD51-recruitment and resistance to PARPi and cisplatin compared to full-length protein (108,109). A recent paper has described the obligated requirement of exon 11 and the CC domain for effective DNA resection and proper RAD51 loading, respectively (110), indicating that both regions have non-redundant functions. Future research will need to address the functional role of exon 11 in the regulation of DNA end resection and tumorigenesis.

### Activities of BRCA1 during transcription and replication

Given its role in the many protein complexes involved in DNA damage repair, the BRCA1 protein is considered a safeguard of the genome. Despite the most well-studied function of BRCA1 is its role in HR, it seems likely that genome protection is also dependent on its activities in transcription and replication. It is widely described that failures in these processes are the most common causes of genetic instability. Conditions that hinder the replication machinery and its associated factors lead to replication stress, which produces replication fork stalling or unfinished replication. The persistence of these conditions could lead to ssDNA gaps or even DSBs, threatening genomic stability (111).

The first report showing that BRCA1 is involved in transcription came from an observation where BRCA1 was acting as a transcriptional activator. It was observed that the C-terminus of BRCA1 fused to a GAL4–DNA binding domain was able to activate transcription in both mammalian and yeast cells (112). Several BRCA1 mutations from patients did not show this behaviour, suggesting a possible transcriptional role in BRCA1-mediated tumour suppression (112,113). Follow-up studies found that BRCA1 was able to interact with active RNA polymerase II (RNAP II) (114). The BRCT domains of BRCA1 are able to bind the RNAP II holoenzyme complex through RNA helicase A (RHA, also known as DHX9) (115). Later on, experiments performed with the heterodimer BRCA1–BARD1 revealed that both C- and N-terminal domains were capable to interact with RNAP II (116). One important partner of BRCA1 is p53—also often mutated in cancers—which has been described to regulate BRCA1 levels in response to cell stress (117). Conversely, BRCA1 overexpression has been shown to stimulate the expression of p53 and the transcription factors p21 and GADD45 (118). Interestingly, BRCA1 also interacts with other transcription factors, drawing attention to this role of BRCA1 in its tumour suppression activity. Examples of these factors are estrogen receptor-α, c-myc, ZBRK1, GATA3, STAT1 or cofactor of BRCA1 (COBRA1/NELFB) (119,120). Mass spectrometry (MS) and two hybrid techniques identified other important BRCA1 interactors engaged in transcription such as TONSL, TCEANC, TCEA and the FACT complex (121).

BRCA1 also interacts with components of the splicing machinery to regulate transcript biogenesis in response to DNA damage (122). In this study, BRCA1 was found to interact with the proteins BCLAF, Prp8, U2AF35/65 and SF3B1. Complex formation upon DNA damage led to the recruitment of the splicing machinery to promoters of certain DDR factors such as *EXO1* or *BACH1*, in order to assure the stability of repair factors under DNA damage conditions. These data suggest that BRCA1 is able to regulate splicing of specific transcripts while at the same time being recruited to DNA damage sites to regulate HR (122).

**Figure 3.**
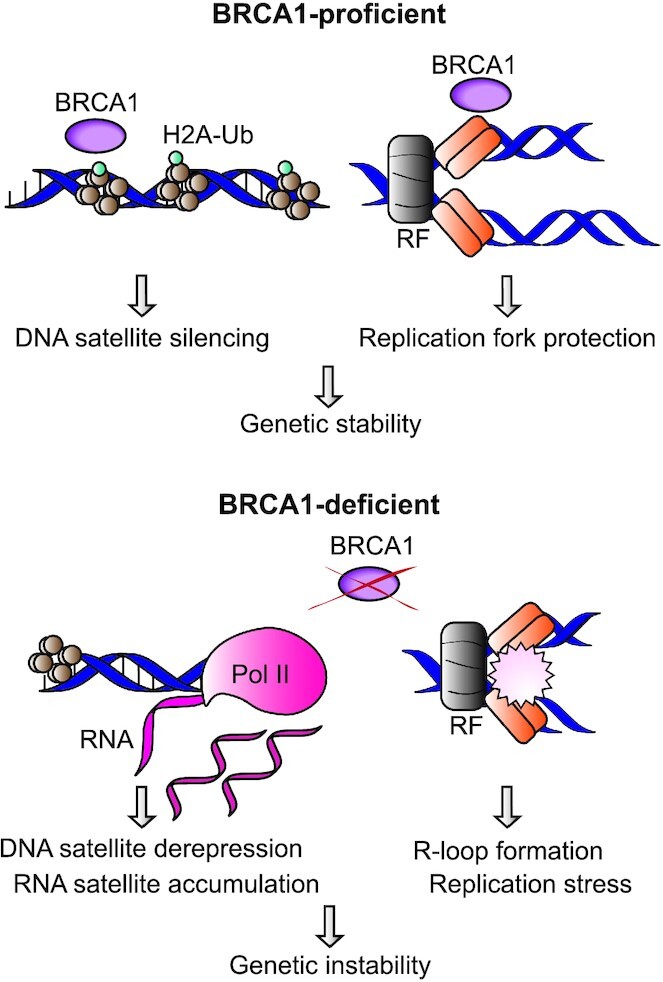
Activity of BRCA1 in the control of chromatin silencing. BRCA1 is important for the ubiquitination of H2A, which controls the levels of satellite transcripts and preserves replication integrity. The absence of BRCA1 enhances the unscheduled transcription of RNA satellites, which leads to the sequestering of BRCA1 partners and formation of R-loops that would hamper the correct replication fork progression (123,124). In addition, BRCA1 is required for the protection of stalled replication forks, preventing degradation and alleviating replication stress.

BRCA1 is involved in the ubiquitination of H2A, which is important for the silencing of DNA satellites (123), apart from being required for HR and resection (92). Zhu *et al.* showed that the absence of BRCA1 leads to loss of H2A ubiquitination and this results in an irregular heterochromatin structure and less HP1 recruitment to these chromatin regions (123). This structural change promotes the expression of heterochromatin silenced regions and increases the number of satellite transcripts or non-coding RNA (ncRNA). It has been shown that these ncRNAs form a threat to the genomic stability in cells (Figure 3) (123). Interestingly, follow-up research showed that ncRNAs in the form of satellite RNAs were able to induce a general DDR and a delay in replication fork progression. Notably, BRCA1 overexpression was able to overcome these replication defects (124). Furthermore, RNase H overexpression partially reduced the replication impairment and the DNA damage accumulation, suggesting that ncRNA-induced R-loops were partially causative for the phenotype.

BRCA1 was first linked to replication fork stability when Scully and colleagues reported that BRCA1–BRCA2 complexes colocalized with nuclear DNA damage induced-PCNA foci in S phase cells (125,126). PCNA, an essential factor for DNA polymerases, interacts with the FA protein FANCD2 to drive repair upon DNA damage (127). Studies performed in *Xenopus* egg extracts showed that BRCA1 unloads the CMG replicative DNA helicase at ICL-induced stalled replication forks to permit the access of other factors for fork processing and repair (128).

DNA fiber experiments showed that BRCA1-deficient cells shortened newly synthesised DNA tracks after hydroxyurea (HU) treatment. Similar results were observed in BRCA2- or FANCD2-deficient cells. These data led to the conclusion that these proteins are involved in the protection of stalled forks by preventing fork degradation (Figure 3) (129). The role of BRCA1 in replication fork protection was also assessed using techniques such as iPOND (isolation of proteins on nascent DNA), permitting the recovery of factors at ongoing or stalled replication forks (130). This tool, combined with MS identification of isolated proteins, showed the presence of BRCA1, and also BRCA1 partners, at HU-stalled replication forks (131,132).

Interestingly, recent data have shown that the interaction of BRCA1 and PALB2 was dispensable for fork protection (94) in contrast to the necessity of this complex for HR and effective checkpoint responses (104,133). In addition, it was found that the phosphorylation of BRCA1 by CDK1 and CDK2 was required for proper fork protection as well as the activity of the prolyl isomerase PIN1. This protein promotes the isomerization of the BRCA1–BARD1 heterodimer, which enhances the interaction with RAD51 during replication fork protection (94). RAD51 forms a filament to protect stalled replication forks from nucleolytic degradation by MRE11, preventing the formation of ssDNA gaps during replication, independently from its role in HR (134). These data show that BRCA1 has dual, non-overlapping functions in HR and replication fork protection. Daza-Martin *et al.* observed that mutations in BRCA1 affecting fork protection but not HR, occur in malignancies (94). However, the significance of these specific mutations for tumour formation and progression has not been interrogated yet.

Mouse studies on BARD1 separation of function mutations have indeed shown that the role of fork protection in tumour formation is still under debate. It has been shown that the BRCT domain of BARD1 is essential for replication fork protection, given that mutations in the BRCT domain of this protein lead to degradation of the stalled fork as well as an increase in DNA breaks upon HU treatment (135). The authors observed that BRCT mutations in BARD1 had no effect on HR in contrast to the BRCT domains of BRCA1, which are required for both HR and replication fork stability. Importantly, mutations in the BRCT domain of BARD1 did not lead to an increased tumour formation in mice, unlike BRCA1 BRCT mutations (135). Therefore, it remains unclear whether the role of BRCA1–BARD1 in the protection of replication forks is part of its tumour suppressor activity.

## R-LOOPS IN THE DNA DAMAGE REPAIR RESPONSE

### The function of non-coding RNAs in DNA repair

The role of small non-coding RNAs in DNA repair and genetic stability have been studied extensively and this topic has been well reviewed (136,137). Preliminary observations in *Saccharomyces cerevisiae* showed that small RNAs are required for efficient repair of a chromosomal DSB, contributing to genomic integrity maintenance (138). Later on, it was shown in human, mice, and zebrafish cells, that DROSHA and DICER RNases are essential for the recruitment of 53BP1 to irradiation-induced DNA damage sites. These proteins are involved in the production and processing of small RNAs upon DNA damage—termed DDRNAs (DNA Damage Response RNAs). The abrogation of the DDR in the absence of DROSHA or DICER could be rescued upon the addition of small RNAs, suggesting an active role of these molecules to preserve accurate DNA repair (139). Similar findings were observed in *Arabidopsis thaliana* (140). Using reporter assays for DSB repair, the authors showed that small RNAs were synthesized in the vicinity of the DSB and were required for correct repair. Interestingly, no changes in γH2AX accumulation after DNA damage were detected upon the depletion of the RNA processing factors AGO2 or DCL3 (140) or DROSHA and DICER (139). In contrast, DROSHA or DICER-depleted cells did show reduced recruitment of phosphorylated ATM, MDC1 and 53BP1, suggesting that small RNAs are playing their role downstream of H2AX phosphorylation during the DDR (139,141).

While assessing whether the generation of DDRNAs resulted from a preformed mRNA or from newly synthetized RNA, it was shown in plants and human cells that transcription emerged upon DNA damage in the vicinity of an induced DSB (140). Michelini *et al.* confirmed this observation demonstrating that damage leads to the local transcription of so-called damage induced long non-coding RNA (dilncRNAs) (142). Using a reporter system in human cells based on the endonuclease *I-SceI*, which is able to induce a specific DSB in the DNA, they observed bidirectional transcription of these dilncRNAs as well as a clear interaction of 53BP1 with the dilncRNAs. The authors also detected an interaction between the MRN complex and RNAP II after DNA damage by co-immunoprecipitation assays. Importantly, MRN binding was necessary for RNAP II and dilncRNAs accumulation (142). Taking together these observations, the authors proposed a model in which there is *de novo* transcription from and to the break site upon break induction, generating dilncRNAs that are processed by DROSHA and DICER to yield the small DDRNAs. The findings indicate that downstream DDR proteins could be recruited to the breaks via these dilncRNAs and the transcription machinery, while upstream DDR factors are important for recruitment of the transcription machinery (142). More recently, a biochemical study using an *in vitro* transcription assay has reported that the ability of MRN to melt DNA ends, and not its nuclease activity, is important to drive dilncRNAs transcription and that the resulting transcripts seem to form transient RNA:DNA hybrids (143). However, additional work will be needed to support these findings *in vivo*.

### The role of R-loops in DSB repair

During the search to elucidate the mechanism by which DDR pathways involve RNAP II activity, multiple groups found an unexpected role for R-loops. Britton *et al.* observed that a bacterial catalytically inactive RNase H localized to damaged regions in human cells. This was dependent on transcription, suggesting the formation of an R-loop upon DNA damage (144). Importantly, this R-loop seems to hamper correct repair as it has been shown in budding yeast that depletion of both RNase H1 and H2 leads to an increase of residual Rad52 foci, indicative of persistent breaks (145). The Fischer lab reported in *Schizosaccharomyces pombe* cells that RNAP II was recruited to a *P-poI* induced DSB and using DRIP-experiments, they unambiguously showed the presence of R-loops surrounding the break site. Interestingly, the authors showed that both overexpression and deletion of RNase H enzymes impaired the correct completion of the HR pathway, suggesting the necessity of a temporary R-loop near the DSB site in order to guarantee the repair (146). Moreover, U2OS cells depleted for the RNA unwinding helicase DDX1 showed an increase in hybrid accumulation at both sides of an induced DSB, indicating that this protein is involved in the hybrid resolution near the DSB region. DDX1 depletion resulted in decreased HR efficiency but had no significant effect on NHEJ (147). Altogether, it seems reasonable that hybrid formation, but also its correct removal, are important steps during break repair and specifically during HR.

Remarkably, there is evidence that transcription-dependent HR occurs in the G_0_/G1 phase of the cell cycle in post-mitotic neurons (148). In this work, the authors postulate R-loop-dependent RAD52 recruitment to DSBs to guide RNA-templated HR in these cells (148). Although this hypothesis is compelling, and studies in yeast and human cells have shown usage of RNA-templates for HR (138,149,150), the physiological relevance of this mechanism and the role of R-loops herein needs to be studied in more detail.

Counterintuitively, there is an emerging body of evidence suggesting that R-loops could be generated as a consequence of the transcription shutdown that is induced upon DNA damage (reviewed in (151)). Indeed, it has been shown that pausing of the RNAP II close to the sites of DNA damage contributes to R-loop accumulation (29,152,153). There are different possibilities that could explain the arrest of RNAP II and the consequential increased R-loop levels. It has been reported that ATM kinase can inhibit RNAP II elongation at distal regions of the DSB possibly via regulation of H2A ubiquitination (154). Also DNA-PKcs has been described to be responsible for the RNAP II eviction when the DSB occurs within the gene body, hindering transcription (155,156). Furthermore, there are also transcription factors (such as TLP1, NELF and ENL), chromatin remodelers (cohesin, SWI/SNF or CDYL1 amongst others), and histone marks such as methylation or ubiquitination, involved in the silencing of the chromatin after DNA damage (157).

To allow the detailed study of the spatial DSB recruitment of repair proteins and chromatin modulation, the Legube laboratory designed a U2OS human cell line with an inducible *AsiSI* nuclease allowing a timely induction of 100–150 DSBs in the human genome (the so-called DiVA system for Damage-Induced Via *AsiSi*) (158). This tool enabled genome wide profiling of the R-loop distribution upon producing these specific breaks. This study showed a correlation between the induction of DSBs and a significant increase of R-loop formation near the break sites (153). Exhaustive analysis of these DRIP-seq data at highly transcribed regions with or without cleavage (159), showed that there is a loss of R-loops in regions up to 100 kb surrounding the break, yet a significant gain of hybrid signal at the actual break position. This is in accordance with a possible effect of the transcription shutdown on a larger window surrounding the break site. However, the clear increase of hybrids at the specific cleavage site strongly suggests *de novo* R-loop formation at this site.

More recently, it has been found that the RNA strand in the R-loop can be methylated, regulating HR activity (160). This highlights another level of modifications that can modulate R-loop homeostasis. It was observed that the ATM kinase phosphorylates the RNA methyltransferase METTL3 after break induction, promoting methylation of the RNA molecule at 6-nitrogen of adenosine (m6A). The authors described that the recruitment of HR factors, such as BRCA1 or RAD51, were highly impaired when METTL3 is absent. With this work, the positive role of R-loops in the repair process is further strengthened.

Another genome wide study, this time in *S. cerevisiae* (161), also supported the idea of R-loops aiding to the repair function. Here, the authors compared R-loop distribution using data from a previous DRIP-seq analysis (162) and Rad52 ChIP-seq data along the genome. It was inferred that R-loops could be classified according to their function and their chromosomal context, determining that a subset of R-loops could lead to DNA damage, whereas a different subset of these structures is essential to modulate DNA repair. Regarding the latter, there are also contrasting observations. In fission yeast, R-loops do not seem to be involved in the repair of certain types of breaks (163). The authors reached this conclusion based on the fact that an RNase H1 and H2 mutant did not show R-loop enrichment at the induced DSB region and repair of IR-induced breaks occurred efficiently in these mutants, contrasting the R-loop-dependent repair findings.

Altogether, these observations lead to a conclusion that there are different types of R-loops. Depending on their nature or context, they could accumulate aberrantly, giving rise to recombinogenic DNA damage and obstruction of replication (reviewed in (67,164)), or perform an essential role in DNA repair. The mechanisms by which R-loops modulate repair remain unclear, but the data suggest that they recruit repair factors to orchestrate the correct repair pathway. However, more work is needed to understand the repair pathways and the role of the R-loop herein.

## FUNCTIONAL INTERACTIONS BETWEEN BRCA1 AND R-LOOPS DURING DNA DAMAGE

Several reports in the last few years have linked BRCA1 to R-loop formation during HR repair and also found implications for disease development. More specifically, it seems that BRCA1 can interact directly with the hybrids but also indirectly acts together with other factors to help R-loop dissolution and the repair process.

Ewing sarcoma cells are characterized by significant DNA damage and R-loop accumulation (165). The analysis of genome wide maps of R-loops, gene expression, and distribution of RNAP II and BRCA1 revealed accumulation of BRCA1 in transcription complexes at R-loop regions in this specific cell type. After DNA damage, these cells could not suppress transcription compared to control cells and this was accompanied by an aberrant retention of BRCA1 at the transcriptional regions with elevated R-loop levels. This resulted in a lack of BRCA1 at DNA damaged regions and as a consequence, an impairment in HR (165).

The relationship between BRCA1, transcription regulation, and R-loops has been studied in more detail in MYCN-amplified human neuroblastoma cells (166). In these cells, MYCN activation leads to an increase in transcriptional elongation. The authors showed that this is accompanied by recruitment of BRCA1 to promoter proximal regions. The comparison of different genome wide recruitment data of RNAP II, MYCN, and BRCA1 led to the conclusion that BRCA1 aids to suppress R-loop formation caused by the RNAP II stalling at transcriptional paused sites, thereby promoting pause release of RNAP II (166). Both papers point to a specific role of BRCA1 in transcription regulation, which seems mutually exclusive with its role in DNA damage repair. Misregulation of transcription, therefore, might attenuate BRCA1’s role in HR, although MYCN-amplified neuroblastoma cells do not show an increase of unrepaired DSBs (166).

Another study showed that reactive oxygen species (ROS) induce R-loop formation and DNA damage in transcribed regions, which are recognized by CSB and RAD52, driving HR-mediated repair. However, in this case, the recruitment of BRCA1 was not necessary for repair, since RAD51 foci formation was not affected by the knockdown of BRCA1 (167). The differential BRCA1 recruitment to R-loops at specific genomic regions suggests that BRCA1 is required for a certain subset of R-loops and raises the question how this protein is recruited to this subset.

One role of BRCA1 in R-loop suppression or dissolution might be related to its association with Senataxin, a large protein of about 300 kD consisting of an N-terminal domain that interacts with other proteins and a C-terminal domain with helicase activity (65,68,168,169). Mutations in the Senataxin gene cause serious neurological diseases: recessive ataxia with oculomotor apraxia type 2 (AOA2) and autosomal dominant amyotrophic lateral sclerosis (ALS) type 1. Interestingly, downregulation of this protein has been associated with cancer susceptibility, suggesting that Senataxin may act as a tumour suppressor (169). Senataxin functions in diverse processes, such as transcription termination of mostly non-protein-coding RNAs (64) but also some mRNAs (170).

Senataxin also plays a role in replication as studies in Sen1 depleted yeast mutants proved that the lack of Sen1 promotes an S phase delay. More specifically, Senataxin accompanies the replisome through highly transcribed genes by preventing R-loop formation in these regions (171) and as a helicase, dissociating RNAP II from the DNA template (65). In addition, a detailed study showed that replication forks encountering transcription are blocked in the absence of Sen1 and the progression of the sister fork of the same replicon is also slowed down (172). Gromak's laboratory was the first to demonstrate that human Senataxin is involved in the resolution of transcriptional R-loops at transcription termination sites. The absence of Senataxin induced transcriptional readthrough and decreased gene expression (173).

A functional relationship between BRCA1 and Senataxin was first observed in immunofluorescence experiments, where a mutant of BRCA1 in mouse cells impaired the recruitment of Senataxin to the sex chromosome, although in this report no clear interaction was observed by co-immunoprecipitation and proximity ligation assay (PLA) (174). Similarly, others have indicated that BRCA1 colocalizes with Senataxin in nuclear foci, suggesting that these two proteins could be collaborating in the same task (175). Furthermore, in another study, Senataxin was found as a clear BRCA1 partner in MS screens (121). Interestingly, the proteins that emerged in this study strengthened the hypothesis that BRCA1 is involved in transcription-induced DNA damage repair and, more specifically, in R-loop-dependent DNA damage repair, given that the interactors FACT (176) and Senataxin (173,177) are proteins involved in R-loop homeostasis.

Senataxin has been shown to be involved in the prevention of transcription-associated DNA damage and forms a direct link between BRCA1 and R-loops. BRCA1 and Senataxin associate to form a complex at highly transcribed regions, and this interaction is needed to avoid R-loop formation at transcription pause sites and consequential DNA damage such as breaks in the ssDNA of the R-loop (178). Follow-up experiments by the Livingston lab have recently suggested that BRCA1—together with RNAi factors—is required for the formation of small single-strand DNA damage-associated RNA (sdRNA) molecules enriched for transcription-regulatory elements. These sdRNAs mediate the recruitment of PALB2-RAD52 complexes which drive the repair of the ssDNA breaks at these transcription-regulatory elements (179). Importantly, these sdRNAs are a new class of RNA molecules involved in DNA damage repair, as compared to the previously described double-stranded DDRNAs described in the previous section (139,140) The authors speculate that sdRNA synthesis and repair is mediated by R-loop formation at such elements, although this has not been formerly proven. Interestingly, the authors show that the complex formation between BRCA1, PALB2, and RAD52 is RNA-dependent (179). Future research will need to show the functional relevance of this RNA-dependency for the function of these complexes in a wider context, such as DSB repair.

Recent work has revealed the importance of Senataxin in the resolution of hybrids upon DNA damage. Senataxin ChIP-seq analyses upon *AsiSI*-mediated DSB induction confirmed that this protein is recruited to R-loop accumulating regions and contributes to their dissolution (153). Depletion of Senataxin led to a higher 53BP1 recruitment and impaired RAD51 foci formation, indicating that Senataxin is important for HR (153). If BRCA1 is the anchor responsible for Senataxin to gain access to the R-loop, it would be expected that there is BRCA1-dependent Senataxin recruitment and both proteins recruit to the same regions upon damage. However, it has not been shown that BRCA1 occupies the same genomic loci where Senataxin is localized upon break induction, so this assumption has to be proven in the future.

In a recent report, it has been shown that laser-induced DSBs in actively transcribed regions induce an R-loop-dependent RAD52 enrichment, which involves BRCA1 in order to remove the RIF1–53BP1 complex that inhibits the HR pathway (180). Interestingly, both transcription inhibition and overexpression of RNase H1 led to an impairment of RPA and BRCA1 foci formation, suggesting that the recruitment of DDR factors requires the formation of an R-loop. Another observation from the same study was that RAD52 knockout cells show a significant increase of R-loops in specific IR-damaged regions, suggesting the importance of RAD52 activity in the R-loop dissolution.

**Figure 4.**
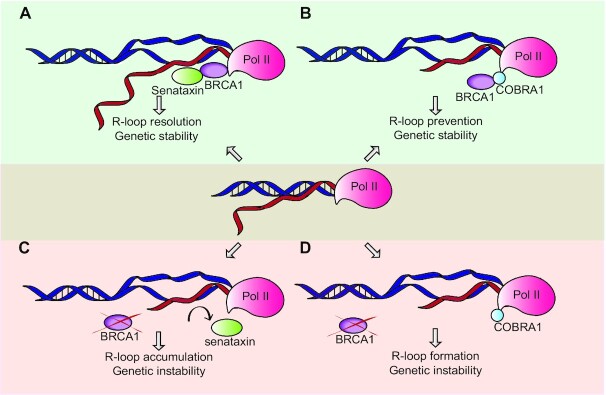
(**A**) Different functions of BRCA1 during transcriptional R-loop formation. BRCA1 can act as an anchor for the correct Senataxin recruitment to permit the R-loop removal and avoid the possible accumulation of genetic instability. (**B**) In addition, BRCA1 can also bind COBRA1 to regulate transcription and diminish aberrant R-loop formation avoiding genetic instability. (**C, D**) Loss of these functions can lead to genomic instability.

Super-resolution fluorescence microscopy (by Stochastic Optical Reconstruction Microscopy: STORM) enabled the visualisation of a direct interaction between R-loop structures and BRCA1 protein, which was highly abrogated after RNase H overexpression (181). Interestingly, the latter did not affect RPA levels at the break, suggesting that the R-loop is generated independently of the step of DNA resection in the HR pathway. This is in contrast with the above-mentioned observation of decreased RPA levels upon RNase H1 overexpression (180). However, both studies agreed on the finding of reduced BRCA1 and RAD51 levels upon transcription inhibition or RNase H overexpression. It was also demonstrated by PLA, IF and co-immunoprecipitation that BRCA2 and RNase H2 enzyme assemble a complex that removes the R-loop and permits RAD51 filament formation (181). Altogether, these results suggest that the R-loop formed at the DSB enables BRCA1 and BRCA2 recruitment. This drives the recruitment of RNase H2, which cleaves the R-loop in a tightly regulated manner (181) to then allow RAD51 loading and termination of the HR process. Thus, the sequence of events seems to reflect that the scheduled R-loop formation at a break must be followed by removal of this hybrid in a controlled manner in order to guarantee correct HR and avoid other secondary effects produced by the R-loop. The described data indicate that RAD52 (180) and BRCA1 (181) are recruited possibly independently of DNA resection and this permits the loading of different proteins involved in R-loop dissolution, such as Senataxin (178), RNase H1 and RNase H2 enzymes (146,181) (Figure 4) or other helicases that bind hybrids (147) to eliminate the transient hybrid and allow for correct repair.

It appears clear that BRCA1 has a role in R-loop suppression, preferentially at termination regions of genes at the 3’ transcriptional pause sites (178). There are also data substantiating that BRCA1 has activity in promoter regions (152,166). A detailed analysis of R-loop distribution along the genome in different types of breast cells showed that those cells bearing BRCA1 mutations tended to accumulate more R-loops than the non-carrier cells and these hybrids were more enhanced at the 5' and 3’ end of the genes (152). This DRIP-seq experiment showed a stronger accumulation of hybrids in the luminal cells than in the epithelial cells of the breast tissue. Interestingly, breast tumours from BRCA1-mutation carriers originate from the luminal cells in the vast majority of cases.

It was previously found that BRCA1 antagonizes COBRA/NELF-B-dependent gene expression during mammary gland development (182). Interestingly, depletion of COBRA1 decreased R-loop accumulation that occurs when BRCA1 is mutated, and this partially inhibited BRCA1-deficiency driven tumour formation (152). Since BRCA1 forms a complex with COBRA1, these data suggest that BRCA1 ameliorates the R-loop accumulation that is caused by COBRA1-mediated RNAP II pausing. This study supports the idea that the role of BRCA1 in R-loop homeostasis is involved in the suppression of tumour formation, but more studies are needed in order to ascertain the potential role of COBRA1 in cancer development upon BRCA1 malfunction.

Taking together these data, BRCA1 could be preventing the accumulation of R-loops together with COBRA1 or aiding in the dissolution with Senataxin (see Figure 4). However, it remains obscure whether the interaction of BRCA1 with Senataxin or COBRA1 implies a different mechanism by which BRCA1 recognises R-loops in the genome and how this could be acting in its regulation for the maintenance of the genetic integrity and the inhibition of tumour formation. One relevant observation that needs to be assessed is that BRCA1 seems to recruit Senataxin to those R-loops prone to induce DNA damage (178), or R-loops formed upon DNA damage (181). Differently, COBRA binds BRCA1 to inhibit the possible R-loop formation during transcription (152), so although the mechanism is unknown, it could be possible that BRCA1 might be promoting the correct RNA processing in this case, similar to the mode of action of the THO complex. More studies are needed in order to test if BRCA1 can also recruit other types of helicases to regulate R-loop formation and accumulation such as AQR, whose depletion showed R-loop accumulation and DNA damage (183,184) or DHX9 (185).

DHX9 has been identified in DNA-RNA hybrid pull-downs and proven to be an important factor in transcription termination and R-loop removal (63), similar to Senataxin (173). As previously noted, BRCA1 interacts with DHX9 to bind RNAP II (115). However, there is conflicting data on whether the interaction between BRCA1 and DHX9 is RNA-dependent (186,187). Overexpression of a DHX9-fragment that binds BRCA1 but lacks its helicase and other functional domains in human cells, led to an inhibition of the normal function of BRCA1, resulting in abnormal cell divisions and a general decrease of BRCA1 foci formation (188). More recently, it has been shown that the BRCA1–DHX9 interaction is increased upon DNA damage and this interaction drives resection of broken ends during HR. However, the recruitment of DHX9 to DNA damage seems to depend on the type of DNA damage, since it only forms foci that colocalize with γH2AX upon CPT treatment but not IR (186). These data indicate the DHX9–BRCA1 complex might be involved in clearance of certain types of DNA damage. Interestingly, DHX9 also seems to have BRCA1-independent roles in R-loop resolution and cell survival. RNF168 has been shown to ubiquitylate DHX9, stimulating its recruitment to R-loops in BRCA1-mutant cells. RNF168 depletion led to a further increase of R-loop levels and reduced tumour formation in BRCA1-mutated cells, indicating a functional role of DHX9 ubiquitylation in R-loop resolution and tumour formation of BRCA1-deficient backgrounds (189). Another link to ubiquitylation has been provided by the identification of the deubiquitylase (DUB) USP42 as an interactor of DHX9. USP42 and DHX9 show epistatic effects on IR sensitivity. Further evidence of a shared function of the two proteins in HR is provided by the observation that depletion of either protein leads to an increase in R-loops upon DNA damage, decreased BRCA1 recruitment to damaged sites, and decreased HR-efficiency (63,187). Currently, it is unclear whether the DUB activity of USP42 is required for its role during HR. However, it is interesting to speculate that there might be a tight functional interplay between the DUB activity of USP42 and E3 ligase activity of RNF168 in the stimulation of DHX9 activity.

Importantly, BRCA1 might not only have a role as an anchor of Senataxin, DHX9 and COBRA for hybrid removal, but also might suppress the formation of ncRNA transcripts capable to form new hybrids (124). The role of BRCA1 in preventing R-loop formation by means of inhibiting unscheduled expression of DNA satellites remains to be clarified. One possibility would be that these transcripts sequester away proteins that normally interact with BRCA1 to protect replication forks. This would then lead to R-loop formation due to the emergence of transcription-replication conflicts. Another possibility would be that the unscheduled expression of RNA satellites directly form R-loops and give rise to DNA damage and genetic instability.

## CONCLUSIONS AND FUTURE PERSPECTIVES

BRCA1 is a multifaceted protein involved in tumour suppression. Strikingly, in spite of years of research, it remains enigmatic how exactly it executes its multiple functions. Recent evidence supports the idea of BRCA1 as a key regulator of the three-stranded structures in the genome termed R-loops. Given the importance of the regulation of these structures for balancing genetic integrity and their unexpected role in DNA damage repair, BRCA1’s function in regulating these structures might represent a novel and underexplored task of its tumour suppressive function. Even more so, BRCA1’s important role in DNA damage repair seems compromised when an increased R-loop load in the cell captures BRCA1 away from the breaks as is seen in Ewing sarcoma cells (165). This indicates that BRCA1 is in a constant limbo to take care of a wide variety of chromosomal disturbances and that a misbalance in one of them might directly affect the other functions of BRCA1. Therefore, we foresee a need for integrative studies on the differential roles of BRCA1 in tumour suppression. In addition to such studies, much can be further explored about the specific role of BRCA1 in R-loop homeostasis. For example, future studies need to address the molecular mechanisms and specific circumstances of BRCA1 stimulating R-loop dissolution together with Senataxin and DHX9 on the one hand and BRCA1 preventing R-loops in complex with COBRA1 on the other hand. Furthermore, studies have suggested that BRCA1 responds to DNA damage-dependent R-loops (181) but also to those that can appear spontaneously (152,178). Thus, BRCA1 could be acting on different types of R-loops promoting their dissolution using different strategies. However, the exact regulation of BRCA1’s function in these strategies is not yet clear.

Interestingly, it has been observed that BRCA1 recruitment depends on the m6A mark in the RNA moiety of the R-loop (160) given that the absence of this mark clearly decreased the correct BRCA1 recruitment to the damaged DNA. It would be interesting to find out if BRCA1 needs this methylation for the recognition of a certain type of R-loop in the genome. It is still unknown whether BRCA1 needs more factors or modifications for the R-loop recognition. The multiple existing BRCA1 protein interactions suggest the possibility of this protein cross talking with R-loops through other proteins or chromatin modifications. However, how BRCA1 and other repair factors are recruited and responding to the R-loops is still a subject of study. Importantly, the new view on the active role of R-loop formation for correct HR repair leads one to wonder whether R-loops are a functional or dangerous structure.
